# Doing nothing? An ethnography of patients’ (In)activity on an acute stroke unit

**DOI:** 10.1177/1363459320969784

**Published:** 2021-01-09

**Authors:** Alessia Costa, Fiona Jones, Stefan T Kulnik, David Clarke, Stephanie Honey, Glenn Robert

**Affiliations:** Wellcome Genome Campus Society and Ethics Research, Wellcome Genome Campus, UK; Florence Nightingale Faculty of Nursing, Midwifery & Palliative Care, King’s College London, UK; Kingston University and St George’s, University of London, UK; University of Leeds, UK; Florence Nightingale Faculty of Nursing, Midwifery & Palliative Care, King’s College London, UK

**Keywords:** inactivity, boredom, stroke, environment

## Abstract

Health research has begun to pay increasing attention to inactivity in its broadest sense as lack of meaningful activity and boredom. Few studies however have taken a critical look at this phenomenon. We explore (in)activity drawing on ethnographic data from observations in an acute stroke unit and post-discharge interviews with stroke survivors and their families. Four themes emerged that explain patients’ (in)activity: (i) planned activities; (ii) ‘doing nothing’, (iii) the material environment of the unit; (iv) interactions with staff. Considering these themes, we seek to problematise received conceptual and methodological approaches to understanding (in)activity. We argue that (in)activity is best conceived not as lack of action or meaning, but as a situated practice encompassing both bodily and mental activities that reflect and reproduce the way in which life is collectively organised within a specific healthcare setting.

## Introduction

Concerns about inactivity among stroke survivors have been raised for more than three decades, with studies consistently showing that patients in acute stroke units spend most of their time inactive and alone (Barrett et al., 2018; Bernhardt et al., 2004, 2008; De Weerdt et al., 2000; Ellul et al., 1993; Esmonde et al., 1997; Keith, 1980; Keith and Cowell, 1987; Langhorne et al., 2011). Rehabilitation research has long recognised the importance of sustained activity, including physical mobility, social interaction and cognitive stimulation (Harris et al., 2014; Parke et al., 2015; Saunders et al., 2014). Environments that offer opportunities for increased frequency and intensity of such activities are therefore considered ideal when recovering from acute stroke (Janssen et al., 2014).

The lack of meaningful activity in hospital is recognised as a problem even beyond rehabilitation. If health research has traditionally focused on sedentariness, a growing body of literature has recently begun to consider the problem of boredom, drawing attention to how the hospital environment can unintentionally deprive patients of opportunities to engage in activities that meet their needs and preferences (Steele and Linsley, 2015). Traditionally considered the preserve of philosophical enquiry (Goodstein, 2005; Heidegger, 1995), boredom is increasingly recognised as a problem affecting patients’ experience and outcomes (Burns, 2017; Steele and Linsley, 2015). It is an experience shared across patients suffering from diverse conditions, from psychiatric disorders (Binnema, 2004; Bowser et al., 2018; Newell et al., 2012; Todman, 2003), to cancer (Inman et al., 2003; Passik et al., 2003), brain injuries (Kenah et al., 2017), end of life conditions (Britton and Shipley, 2010) and stroke (Luker et al., 2015). Whether explicitly or not, the emerging literature on boredom is contributing to broaden the definition of inactivity beyond the narrow focus on physical activity. Placing emphasis on the (lack of) meaning, the concept of boredom re-directs attention to the question of how patients spend time outside of planned care and to the mutual relationship between patients’ actions and emotions.

## The problem of (in)activity

Conceived as something that is not happening, inactivity can challenge research methods designed to capture action and meaning (Friedman, 2012; O’Neill, 2017). Research on inactivity has traditionally privileged a quantitative approach, measuring the duration, frequency and intensity of observable activities. Quantitative studies typically represent (in)activity as a series of snapshots of what people do at a given time (Barrett et al., 2018; Janssen et al., 2014; Shannon et al., 2018). They overlook the fact that, from an experience-near perspective, activity and inactivity are not the sum of on-and-off static moments, nor are they necessarily alternative to each other. For example, one can be busy doing something while feeling bored (Heidegger, 1995), or one can be caught up in the hustle and bustle of everyday life but report to be ‘doing nothing’ if the activities at hand fail to register as meaningful and purposeful (Mains, 2007; O’Neill, 2017). Defining meaningful activity as simply the presence/absence of action can then reproduce received conceptualisation of the problem, while pre-empting a more critical understanding of *why* patients engage in certain activities (or not).

Qualitative studies exploring patients’ experience of inactivity are rare. Where quantitative research prioritises ‘doing’ at the expense of meaning, qualitative studies on patients’ experience often do the opposite, privileging how patients make sense of what they do as if meaning was primarily constructed within the individual’s mind. Existing ones show that patients often report being bored and spending long periods of time waiting for externally delivered activities (Clarke et al., 2018). Evidence indicates that the foreignness of the environment can also contribute to these experiences, to the point where even perceptions of familiar activities are affected whilst in hospital (Cheah and Presnell, 2011). These studies can add complexity to the binary definition of (in)activity as measured by health care researchers, for example by providing insights on patients’ lived experience and how their views of meaningful activity may differ from those of health professionals (Harmer and Orrell, 2008; Peiris et al., 2012). Too often however this type of research tells us little about how patients act (or not), prioritising instead reasoned accounts of patients’ experiences. As such, they reproduce a view of (in)activity as a form of individual behaviour, stemming from the inner motives of self-contained subjects (Cohn and Lynch, 2017).

In seeking to address these gaps, we draw on practice theory and explore patients’ (in)activity as situated practice. Theories of social practice have diverse roots and formulations but share the view that human behaviour is shaped by the routinized and situated practices through which people carry on their life day to day (Shove et al., 2012). According to classic practice theory, the way in which people ‘go on’ through the flow of their daily activities is guided by embodied dispositions, which are acquired through practical engagement with the settings of actions and, in turn, affect people’s perceptions, judgements and actions (Bourdieu, 1977; Giddens, 1984). Practice theory therefore de-centres agency from the mind of the subjects and foregrounds action and the settings in which activity takes place (Reckwitz, 2002). With regard to the latter, recent practice-based approaches have expanded the definition of social settings, previously intended in terms of norms and rules, drawing attention to the materiality of the environment and the ways in which practices are enacted by both humans and non-human actors (Mol et al., 2010). This approach draws attention to the bodily movements and dynamic actions that tend to remain out of sight in accounts of patients’ experience, without reducing activity to a set of static measurements.

Recent praxeological approaches (Gherardi, 2017; Reckwitz, 2017) have also taken a particular interest in affects as a domain that remained poorly theorised within classical practice theory. From this perspective, practices are not just routinised behaviours performed unreflectively; they also encompass mental and emotional activities which guide people’s attention and motivation (Reckwitz, 2002, 2017; Schatzki, 2002). Affect is the concept of choice to denote how such emotional activities do not occur within an individual’s psyche but are embedded into social practices; they are embodied as much as felt, and they exude from and circulate among non-human objects and material environments as much as among human actors (Reckwitz, 2017). While retaining a distinctive focus on situated action, this approach explores practices as affectively attuned, thus paying particular attention to why actors take part in social practices. Practice-based approaches have variously been used to explain health-related practices, from rehabilitation work (Struhkamp et al., 2009) to interventions to increase physical activity (Cohn and Lynch, 2017; Nettleton and Green, 2014). Here, we use practice theory to analyse – for the first time – patients’ inactivity.

## Methods

This article draws on data from fifty hours of observation in an acute stroke unit in South East England, and nine interviews with stroke survivors and family members who received care in this unit. Data collection was undertaken as part of a larger, mixed-methods study evaluating the use of co-production to improve the experience of patients, families and staff in four acute stroke units (Jones et al., 2020).

### Participants

Eight patients and six family members took part in the interviews. The sample included patients with significant disabilities. Five were wheelchair dependent and non-ambulatory and three had a communication disorder (aphasia). Three were under 65 years.

#### Data Collection

Data collection took place between 2016 and 2017. The study received ethical approval from the Health Research Authority (UK) and a Research Ethics Committee (anonymised). Written consent was obtained by participants before the interviews. Unit level permission for observations was obtained before the start of the study. During the observations, we treated consent as a process (Dewing, 2007) and always obtained verbal consent before entering patients’ rooms and/or when circumstances arose which may affect patients’ privacy and confidentiality (e.g. family visits).

AC and FJ independently carried out a total of 50 hours of ethnographic observation (Atkinson and Hammersley, 2007). Observations were unstructured and guided by a practice-based approach, focused on documenting what patients did at different times of the day/week, where, with whom and for how long. They were arranged during weekdays and weekends, typically for periods of four to eight hours between 07.30-14.30/14.30-20.30. Researchers did not take part in the activities observed, whether delivered by staff or initiated by patients, but did engage in informal conversations (inevitably disrupting patients’ inactivity on the few occasions this happened). Fieldnotes were hand-written and anonymised transcriptions were shared with the research team.

AC, FJ and STK conducted a total of nine interviews, including three with patients, one with a family member and five joint interviews with patients/families. Interviews were semi-structured and followed a topic guide exploring: how patients spent their time on the unit, what they did/would have liked to do outside of therapy, what helped or prevented them from doing so, and what improvements they would have liked to see in this respect. Interviewees were recruited through convenience sampling and were first approached and given information about the study by clinical staff on the unit. The interviews took place within six months from discharge and were conducted at participants’ homes or the researchers’ institutions, depending on participants’ preference. The interviews were filmed for the purpose of the wider study (Bate and Robert, 2007); audio-recordings were fully transcribed by a professional transcriber.

### Analysis

Interviews were manually coded and analysed thematically (Charmaz, 1990; Glaser and Strauss, 1967). Codes were initially derived from the topic guide and further refined in an iterative process. Fieldnotes were read multiple times and compared with interviews, paying attention to those aspects of patients’ observed activities that remained unarticulated in interviews to test and expand the themes identified. A summary of our preliminary analysis was discussed with participants during two separate feedback events. FJ, STK and a professional facilitator moderated the discussion, while AC took hand-written notes that were then transcribed and included for analysis in this article.

In line with established principles of ethnographic research (Atkinson and Hammersley, 2007), analysis was iterative, beginning during data collection and carrying on through the writing up of findings. We drew on practice theory because it aligned with our focus through data collection and helped us to address what we saw as conceptual gaps in the literature. Findings are fully anonymized and participants’ names have been replaced by pseudonyms. Excerpts from interviews and field notes have been minimally edited to facilitate reading (edits are indicated by the use of parentheses). Quotes from field notes are reported in inverted commas when presented verbatim.

## Findings

Four themes emerged which explain patients’ (in)activity: (i) planned activities; (ii) ‘doing nothing’; (iii) the material environment of the unit; iv) interactions with staff. The findings below present a summary of the qualitative themes; they do not report on the frequency or duration of the activities discussed –although in these regards, our observations confirmed that patients spent most of the time outside of therapy inactive and alone (Bernhardt et al., 2004).

### Planned activities: ‘It was like sitting in your room, and breakfast, dinner, tea’

When asked to describe what they did on the unit, all our interviewees focused on planned activities delivered by staff. Sometimes, this was the case even when emphasis was explicitly placed on the times outside these planned activities:

Interviewer: Can I ask you to take me step-by-step during one of your average day at the hospital? What were you doing when you had no planned therapy?

Matt: Well, not a lot, well, normal things, wake up, you get washed or dressed, try and have a shave or something like that, or have a shower, get dressed, have some breakfast [. . .], have your tablets. If it’s Monday to Friday, because nothing happened at the weekend at all, doctor will come round [. . .] and nothing, nothing was planned, just carry on, hopefully get a paper to read and that was it I’m afraid.

Planned activities, including mealtime, medical visits and nursing care, were organised in daily and weekly cycles. The staff hand-over at around 7.30 am marked the start of the day. While the ward round was being completed, the domestic staff would arrive with breakfast and patients were woken up to eat their meal and take their medications. After breakfast, patients were assisted with washing and dressing, and their beds were made. During weekdays, the medical ward round would take place. Where assessed as appropriate, patients would be scheduled for physiotherapy, occupational, or speech and language therapy, although they would not always receive the nationally recommended fortyfive minutes of each (Intercollegiate Stroke Working Party, 2016). Depending on their needs and condition, patients might also join the weekly therapist-led groups. Mealtimes and visits from friends and family punctuated the time outside of therapy sessions.

Weekends represented a significant downturn in terms of variety and frequency of these planned activities, as therapists and medical doctors were not on shift:

At the weekend, the only people you would see would be the people that come around and give you your breakfast [or] your drink. [. . .] Then you’d have your lunch, visitors would come in the afternoon, that’s it, there was nothing else to do. [. . .] There was nothing at the weekends, nothing at all. *(Liz)*

Patients often stressed the fact that during weekends ‘nothing happened’. Families and nursing staff also shared this view:

“Nothing happens”, says a Health Care Assistant (HCA). “Weekends are long”, comments another HCA. “Weekdays are very busy – patients have got everyone coming into their rooms. At weekends, they only see us. They get their care, obviously, but that’s it. It’s like – what happens now? Do I count down to Monday?” she says looking down at her wrist. (*Field notes from staff event, 08 March 2017*)

These responses resonate with our observations, showing that patients were indeed more active during planned activities. This was particularly evident at mealtimes. Meals were consumed at bedside and did not offer opportunities for leaving the room or socialising with other patients. Despite this, the routine of getting ready to eat, having food served and exchanging a few words with the domestic or nursing staff meant that most patients who were not extremely unwell appeared significantly more alert and engaged during mealtimes. By contrast, once meals were over, the atmosphere in the rooms quickly returned to deep quiet and even patients who had appeared fully alert only a few minutes earlier could be seen dozing off before the tray on their table had even been cleared.

Planned activities structured patients’ day, providing regular occasions to take part in activities that were, at least to a certain extent, participated by others and oriented towards a common goal. As a result, the very notion of doing something on the unit was synonymous with taking part in planned activities delivered and administered by staff. The long periods of time between and outside these planned activities were often glossed over as a time when “nothing happened” (Matt) and failed to register in patients’ reasoned accounts.

### Doing nothing

Of course, planned activities only made up a fraction of patients’ day. Here we look more closely at what patients did between and outside planned activities, particularly during weekends. Socialising with other patients, friends and family or, when possible, with staff were the most common activities and patients proactively sought opportunities to get to know other people. Other activities that patients discussed included listening to the radio or watching TV, reading books or a newspaper, using colouring or puzzle books. Others described using their time to engage in self-care practices, like getting a shower or receiving a massage. Some said they practiced the exercises provided by therapists.

All interviewees described at least some examples of meaningful activities and acknowledged their benefits in terms of rehabilitation and overall experience. Socialising was seen as an important source of mutual support. Activities that offered the opportunity to get out of bed were appreciated as a source of physical exercise, while pastimes such as reading or listening to the radio were seen as a way to keep cognitively active. Despite this, observations indicate that similar activities were an infrequent part of a patient’s day. For example, while socialising was regarded as both enjoyable and helpful, patients in four-bedded bays could often be observed staring at the wall while being silent in the presence of each other. Similarly, while patients reportedly appreciated therapists’ encouragement to exercise on their own, it was rare to see them doing so.

Interestingly, however, even when patients were apparently doing nothing, they still ‘did it’ in specific ways. Our fieldnotes abound with descriptions of small, repetitive and inconsequential actions, such as picking up and putting down a book without reading it, peeking out to the corridor as someone passes by without striking up a conversation, going though one’s personal belongings without using them. One of the most common scenes one would observe on the unit was that of people lying in bed seemingly asleep. If one observed long enough it would become apparent that patients were not really asleep. They alternated between trying to doze off and staring into the void, just waiting for time to pass. In fact, one of the paradoxes of observing people being inactive was that the absence of action, like when one is really asleep, could in fact mean that the person was engaged in meaningful activity by getting some rest. On the contrary, the repetitive and inconsequential small actions we often observed suggested patients were just trying to kill time when there was nothing to do.

On the one hand, the difference seems obvious between ‘doing nothing’ and the examples of meaningful activities that patients described, such as socialising, reading or playing games. On the other hand, however, the observed experience of ‘doing nothing’ resonates with patients’ description of meaningful activities. Patients talked about these activities as a source of “distraction” (Martha, Julia’s daughter) and a way to “fill our time up” (Trevor). They said that engaging in some form of activity outside of planned therapy helped them to “break up the monotony of the day” (James) and provided an escape from the “soul destroying and boring [prospect of] just sitting there all day and don’t do anything” (James). In other words, they described meaningful activities as a way to help pass time:

Some people play cards and I like cards, I would have liked a card game. I think *it takes a lot of hours off your life*. (*Danielle, emphasis added*)

The time didn’t drag for me because I listened to the radio. For some reason there was usually something to do like a blood test or an ECG. The time didn’t drag because *I was focused on leaving [the unit]*. (*Ian, emphasis added*)

While patients’ own distinctions between what they considered helpful or enjoyable (or not) are important, the findings indicate that patients often did not engage in the activities they described as meaningful, suggesting that their views on different types of activities do not fully account for their actions. At the same time, the findings also show that when they said to be doing nothing, patients did not simply ‘switch off’ but tried to engage in some sort of occupation to pass their time (cf. O’Neill, 2017). Meaningful activities and ‘doing nothing’ were accounted for in almost opposite ways: the first were remembered and valued, while the second failed to register as meaningful or worthy of mention. At the level of practice, however, both experiences were characterised by a sense that time was at best passed or killed because there was not much to do.

### The environment: ‘Not as though I’ve been in prison, but. . .’

**Figure 1.**
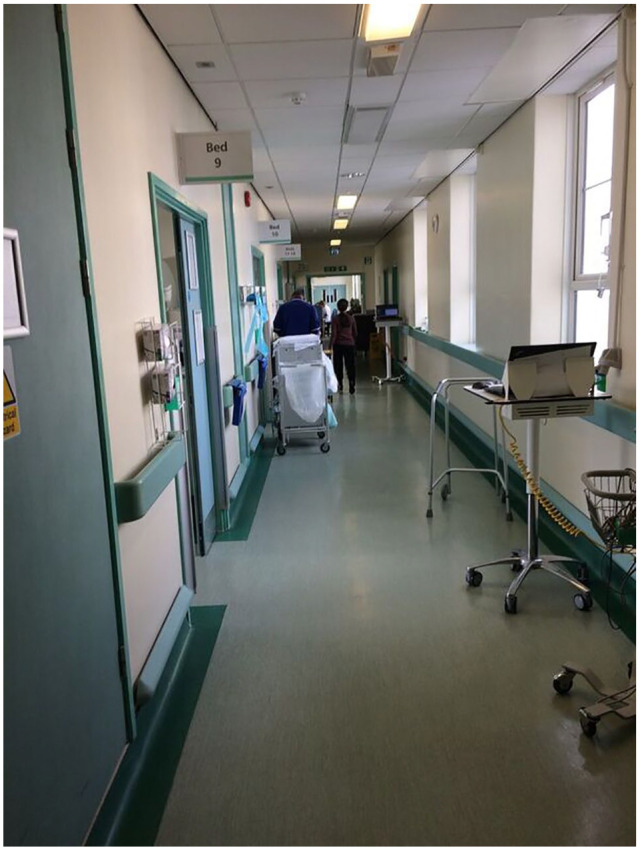
The corridor with patients’ rooms on the left.

The unit was organised into two sections laid out in an L-shape, with a total of four four-bedded bays and eight single rooms. Both sections were similarly designed, with rooms and bays lined up on one side facing onto a corridor. Other areas patients had access to included the gym, which remained closed when therapists were not on shifts, the corridor and an open space, which typically served to store large pieces of equipment. The corridor was similarly cluttered with chairs, desks, trolleys, filing cabinets and various pieces of equipment (Figure 1), which made it prohibitively narrow for patients who used an aid to walk and just wide enough for one fully mobile person to move at a time. The various objects located in the corridor were an integral part of staff’s work, meaning that even during the quietest times these areas usually buzzed with staff filling out notes, moving equipment, making telephone calls, managing patients’ admission and discharge, catching up with colleagues or patients’ families. Contrary to this, one thing an observer would immediately notice was the absence of patients in communal areas.

Concerns about safety significantly constrained patients’ movement, both within and outside the unit. Interviewees usually showed awareness at the limitations and precautions they had to take to avoid falls or injuries, but did not recall being encouraged to leave their room. For the majority of patients who were physically impaired, movement to different areas of the unit and hospital was almost entirely dictated by staff and visitors. Even patients who were fully mobile had to create their own opportunities to take a walk, such as using the toilet furthest from one’s room. The unit offered little incentive for them to be out of their bed, let alone space to move comfortably around.

Patients who were physically or cognitively impaired were actively discouraged from attempting unaccompanied access to areas of the hospital outside the unit. The absence of staff or trained volunteers to escort them limited their opportunities to leave the unit and be in a more familiar, non-medical environment:

Richard [Liz’s husband]: Liz’s best relief was on a Saturday when I used to take her down to the canteen you know because. . .

Liz: [We] used to go down to the restaurant on a Saturday and we’d have coffee and a cake.

R: Yeah, see civilisation.

L: If the weather was nice we used to go and sit outside the hospital and he’d go to the shop and get us an ice cream.

Interviewer: How did these activities help you?

L: Oh, a lot.

R: It was a release.

L: Yeah, it used to get me out of the ward, to go downstairs, it was really nice. In fact we were sitting out the hospital one day and a bus went by, and I said, ‘Oh, quick, there’s a bus! Let’s get in and go home!’ [Laughs]. ‘Let’s escape!’

Inside the unit opportunities for recreational activities were scarce. Each bed had a radio and television; the latter was available free of charge only in the morning and most patients reported not using the service outside of these hours because of the high costs. A trolley would bring newspapers and other basic items from the shops, but the service was sporadic and irregular, and entire weekends could go by without the trolley being seen:

I wasn’t allowed out of the ward. I was on the third floor, I think, and sometimes the shop wasn’t open. I had to wait ‘til people [came to visit] so it’s probably in the afternoon that I could get [a newspaper]. But I like it early in the morning, then you know what’s going on. I didn’t use the television [either]; it’s very expensive in the hospital and I couldn’t afford that. [. . .] They could have come around with more books, couldn’t they? Like a Prison Service, they give you books don’t they, come around! Not as though I’ve been in prison, but they give you a book, something to look at! (*Matt*)

**Figure 2.**
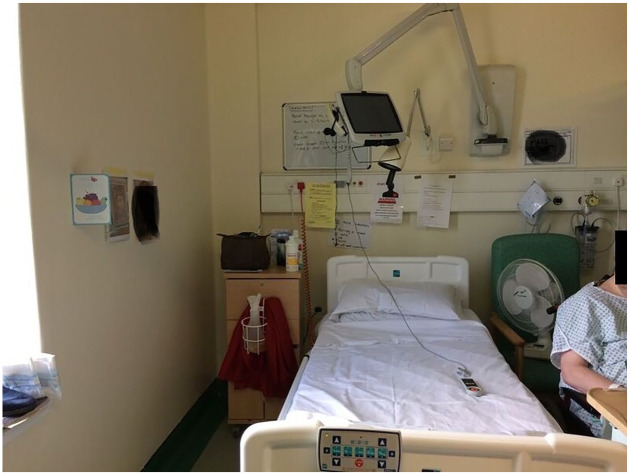
The area around the patients’ bed.

Apart from the radio, TV and occasional newspaper, the environment of the unit was very clinical. The gym was the only space with dedicated equipment for patients to practice activities; other objects which could provide opportunities to keep active, such as games, magazines or art and craft tools, were not available in other areas of the unit. The bed areas were also typically rather bare (Figure 2). Personal belongings were stored in the small cupboard near the patient’s bed; only a few items for everyday use were kept at hand, such as eye-glasses, some snacks, a book or a cardigan that could be worn over the hospital gown that many patients wore throughout the day.

One of our interviewees, who tried to decorate her mother’s room, noted how the lack of personal belongings was extremely de-personalising:

The one thing I really wanted was to make the staff understand who she was, so I took with me lots of photos of the family, of the weddings, of mum with friends [. . .] and I was putting them all round the walls just so people would get to know my mum as the person, not my mum as the stroke sufferer. (*Martha, patient’s daughter*)

The patient similarly explained that the pictures helped her to keep cognitively active by giving her something to focus on. Moreover, they facilitated socialization with members of staff, offering prompts for conversation around topics that were not limited to the basic routines of hospital life. in hospital. This form of activity was also a way to perform and protect her identity: “I could talk about my children [. . .] and I felt it helped reassure me that I was still somebody” (Julia).

Patients’ statements and our observations on the unit resonate with existing evidence highlighting the impact of restrictive and impersonal environments on patients’ activity and boredom (Bowser et al., 2018). The unit was seen as a closed world, to the point where it was compared to a ‘prison’ or a place from which one wished to be ‘released’. Similar to prisons and other total institutions (Goffman, 2017), being a patient on the unit was also characterised by a loss of personal identity which both contributed and reflected the lack of personal belongings and familiar occupations.

### Interactions with staff: ‘You didn’t know how long that minute would be’

Interviewees valued interactions with professionals as a source of support as well as distraction, however they also perceived staff to be over-stretched and short of time to provide the support they needed to remain active. Some interviewees felt that staff were too busy to assist them undertaking daily activities at their own pace and would rather complete tasks on behalf of patients, thus failing to appreciate patients’ potential and encourage them to be more independent. Others commented that staff were often unavailable when needed, leaving them waiting without knowing when things would be done.

This sentiment most usually referred to nursing staff. Overseeing continuous care throughout the day, nursing staff were crucially placed to ensure that patients could initiate and sustain autonomous activity, as illustrated in the following vignette:

At bed 21, the curtains are closed. Behind, I hear nurse Sally talking with a man. “Do you really need a pad Nick?” The board next to the bed reads: “please mobilise to toilet.” “Do you accidentally wet yourself?” asks Sally. “I’d encourage you to try to be continent rather than using the pad. Pads are really not good for you.” I hear Sally helping Nick sitting in his chair and getting dressed. She invites him to wear his trousers and encourages him to do it on his own: “I want to see how much you can do for yourself. Well done!” Sally then asks Nick about shaving -does his wife helps him at home? Does he use an electric razor? Nick says he does his shaving by himself at home, but he also lets his beard grow sometimes. Sally says she’ll look to find him a razor [. . .] “Do you want your TV on Nick?” asks Sally. “No.” says Nick. Sally leaves promising again she’ll look for the razor. (*Fieldnotes 14.01.2017*)

As shown, nursing staff played a key role in monitoring whether patients were as active as they could be. Nurses’ interactions with patients were also essential to explore and address patients’ unique needs and capacity in practice. This type of information was displayed at each bed, including details on patients’ levels of dependency and need for assistance. Therapists also used coloured posters to share information on how to support patients’ activities by facilitating communication for patients with speech impediments, providing appropriate stimulation, or monitoring movements for patients with physical disabilities. The information was directed at staff and not easily accessible to patients or visitors: the tools used specialist jargon and were not necessarily visible from the patient’s own bed (i.e. they were positioned behind the bed head). Interactions with nurses were crucial to share this information with patients, ensure that they had been communicated and remembered what they could do on their own and explore what prevented them from doing so.

Nurses’ intervention was also key to facilitate patients engaging with the environment, as highlighted in the vignette where the nurse repositions the TV so that the patient can view it. Other examples that patients discussed included: getting a bed pan in time to be continent, having the lid of the yogurt pot peeled off or getting help to put dentures in to eat independently, having fresh water positioned with the handle turned towards the able side of the body; and receiving help to plug a mobile telephone into a charger that was positioned behind the bed. These material arrangements were not just a matter of comfort; as discussed, they could enable or prevent patients from remaining autonomous and engaging in activities on their own. Having to repeatedly ask for assistance when these things were not in place, on the other hand, could bring to consciousness patients’ reduced autonomy.

In the vignette, (some) of this work is undertaken whilst the nurse helps the patient get dressed for the day. Contrary to this, observations indicate that most of the time staff’s interactions with patients remained focused on the specific task at hand. Standing in the corridor one would observe staff moving in and out of rooms and bays for just the time it took to complete a task before moving on to the next one. Often, this would happen without patients and staff speaking to or interacting with each other. Communication was kept friendly, but nurses were careful to manage interactions with each patient, completing the task for which they had entered the patient’s room and deferring other requests. Even interaction that seemed friendly could often appear routinized and impersonal when observed over time.

The routinisation of clinical work around the completion of distinct tasks, coupled with the chronic shortage of staff, meant that despite being available information about each patient’ unique needs and capacity for activity was not embedded in staff’s work practices. When this did happen, it was mainly in response to patients’ specific requests for assistance or as part of routinized interactions, such as when staff would ask patients if they wanted to be fed or eat independently at meal time. Outside of these occasions, interaction with patients was mainly time-bounded and task oriented, leaving little room to explore and address what patients could and wanted to do, what possibly stopped them and what could have helped.

## Discussion

We have analysed patient’s (in)activity, focusing in particular on what patients did outside of planned routines. We have shown that patients ascribed meaning to activities not (only) because of the content and type of the activity itself, but because of the way in which doing something helped them to fill in time and stave off boredom. Despite this, we have observed that patients often did not engage in the activities they described as meaningful, such as socialising, reading, playing games or doing the exercises provided by therapists. Instead, they spent long periods of time ‘doing nothing’. ‘Doing nothing’ was characterised by distinct ways of acting, which did not suppose purposeful intention on the part of patients, but were nevertheless oriented towards a practical aim and adjusted to patients’ necessities (Bourdieu, 1977; Schatzki, 2002). Like reading or playing games, it appeared as a solitary effort to pass time when there was not much going on. These findings are largely in line with existing evidence on high levels of inactivity among stroke patients and in-patients more generally (Bernhardt et al., 2004). Moreover, they resonate with the literature describing patients’ experience of inactivity in terms of apathy (Harris et al., 2014), passivity (Clarke et al., 2018) and boredom (Steele and Linsley, 2015).

Few studies however have taken a critical look at these concepts, focusing either on impersonal, measurable patterns of behaviours or on patients’ subjective experience of being (in)active. Our analysis challenges this kind of conceptual approach, indicating that inactivity was not simply equivalent to the absence of action, nor was it the outcome of patients’ inner views and motives. In making sense of these findings, we have drawn on practice theory to conceptualise (in)activity as a situated practice, that is to say as a bundle of bodily as well as mental activities coherently orchestrated in ways that both reflect and reproduce how life was collectively organised on the unit (Reckwitz, 2002). We argue that this approach can help to link patients’ actions and their experience of (in)activity, explaining why inactivity remained such a prevalent feature of their time in hospital despite being described as frustrating and even “soul-destroying”. Specifically, our analysis has highlighted three ways in which the environment of the unit affected patients’ (in)activity.

First, a growing literature is examining the role that the hospital environment unintentionally plays in depriving patients of opportunities for keeping more active. With regards to stroke rehabilitation, studies have focused on the influence that environmental enrichment may have on increasing activity of stroke survivors, and how increased activity may, in turn, be a factor in improving outcomes such as engagement in rehabilitation therapies, social engagement, improved mood and enhanced confidence (Janssen et al., 2010, 2014; Khan et al., 2016; Rosbergen et al., 2017). Adding to this line of enquiry, we have shown how the material environment is not just a background to patients’ activities but directly shapes patients’ actions and experiences. Physical spaces can contribute to enabling and sustaining patients’ movement, or may limit and constrain it. Environments also have affective qualities. In particular, we have highlighted how the lack of personal objects contributed to reinforcing patients’ perception that meaningful activities had no place in the unit. While it is well recognised how personal objects can help preserve one’s sense of self in practice and resist becoming just a passive recipient of others’ care (Goffman, 2017), our analysis has shown that the role of objects is not merely symbolic, but also performative in that they help to embed activities into patients’ daily routines.

Such material arrangements however are never stable (cf. Cohn and Lynch, 2017; Mol et al., 2010): dentures need to be made available in the morning, TVs need to be repositioned after beds have been re-made, and mobile phone need to be recharged. The second contribution of practice theory is therefore in explaining how the capacity of the environment to affect patients’ activity is not the quality of any object alone, but a function of how the environment is engaged with in ways that are always inevitably local and contingent. In these regards, practice theory has enabled us to situate patients’ (in)activity within the organizational culture of the unit. Specifically, we have shown how the practical organisation of staff work, as itself a form of routinised behaviour (cf. Purkis, 1996), effectively directed their attention away from patents’ unique capacity for activity and from the contingent material arrangements on which this depended.

Finally, we have shown that patients’ own activities were largely subsumed into the daily and weekly organizational arrangements of the unit. This resonates with Goffman’s famous description of total institutions, where every aspects of daily life is organised through a series of activities enforced from above and planned to ensure the efficient achievement of institutional care (2017). The main quality of the time outside these planned routines was that it remained largely unspoken about in patients’ narratives, suggesting that the unit organisational arrangements were also internalised. In other words, it was not just that nothing happened outside of the planned routines of mealtimes, therapy and medical care; nothing was expected to happen. Here, practice theory helps us to move beyond the individualistic connotation of experience to consider how moods and emotions come to be experienced, reinforced and incorporated through the process of taking part in in situated practices (Reckwitz, 2017). From this point of view, the organisational arrangements of the unit did not only structure patients’ patterns of behaviour, but also shaped what made sense for them to do. This view helps explain the lack of focus which characterised the time outside of planned routines, when meaningful activity unravelled into ‘doing nothing’ and even patients who appeared fully alert only a few moments earlier could be seen dozing off in their beds. It also explains why it was difficult for patients to keep active when motivation to do so mainly came from a desire to avoid boredom or, at best, improve clinical outcomes.

### Limitations

We acknowledge that there are limits to retrospective accounts of practice and observations (for example, it may be ultimately impossible to know if a person lying in bed with her eyes close is dozing off or practicing some cognitive exercise). This was not a significant limitation for this type of study, as our aim was not to accurately discern every activity that patients did at a given time but rather to explore how (in)activity became embedded in patients’ routines. The use of convenient sampling was also a limitation. Future research might further explore how (in)activity might be different for patients who are differently affected by the stroke.
